# Three-Dimensional Displacement of Upper Cervical Vertebrae in Severe Mandibular Deviation Caused by Condylar Hyperplasia: A Tomographic Segmentation Study

**DOI:** 10.3390/diagnostics16040579

**Published:** 2026-02-14

**Authors:** Claudia Milena Ramírez, Rodrigo Cárdenas-Perilla, Luis Eduardo Almeida, Diego Fernando López

**Affiliations:** 1 Orthodontics Department, Universidad del Valle, Cali 760043, Colombia; claudia.milena.ramirez@correounivalle.edu.co; 2 Nuclear Medicine Department, Clinica Imbanaco-Grupo Quirón Salud, Cali 760001, Colombia; 3 Surgical Sciences Department, School of Dentistry, Marquette University, Milwakee, WI 53233, USA

**Keywords:** cervical vertebrae, condylar hyperplasia, mandibular deviation, x-ray computerized tomography, imaging three-dimensional

## Abstract

**Objective**: To evaluate the three-dimensional (3D) angular displacement (Roll, Yaw, and Pitch) of the upper cervical vertebrae (C1, C2, and C3) in patients with severe mandibular deviation (MD) due to condylar hyperplasia (CH), utilizing a computed tomography (CT)-based segmentation approach. **Methods**: This retrospective cross-sectional study included 50 patients with MD ≥ 6 mm caused by hemimandibular elongation (HE) or a hybrid form (HF) of CH. The skull, mandible, and cervical vertebrae (C1–C3) were segmented using 3D Slicer software. Angular deviations (Pitch, Yaw, Roll) were measured relative to the Frankfurt plane. Patients were categorized by the side of CH (right or left), and intergroup comparisons were performed using Kruskal–Wallis and Mann–Whitney U tests. Spearman’s correlation analyses assessed associations between MD magnitude and cervical angles. **Results**: CH was significantly more prevalent in females (58%; *p* = 0.021). C2 and C3 exhibited significantly increased lateral Roll inclination toward the side of deviation (*p* = 0.006 and *p* = 0.045, respectively). C2 Pitch negatively correlated with MD severity bilaterally (r ≈ −0.51, *p* = 0.02 right; r ≈ −0.50, *p* = 0.02 left). Strong intra-vertebral correlations between Pitch and Yaw were observed in C1 and C2, indicating synchronized vertical and rotational motion. No significant intergroup differences were found in Yaw angles (*p* > 0.05). **Conclusions**: Patients with CH and severe MD exhibit consistent patterns of 3D cervical displacement, particularly in lateral inclination and vertical movement, suggesting compensatory postural adaptations in the upper cervical spine.

## 1. Introduction

Condylar hyperplasia (CH) is characterized by overgrowth of the mandibular condyle, which disrupts normal facial development. Histologically, this condition presents as an increase in cell number and thickness within the soft condylar layers [1,2]. This overgrowth can lead to severe deformities because it can occur not only during growth and development but also in adulthood [3]. Depending on its duration, type, and severity, CH can affect the temporomandibular joint (TMJ) structures (condyle, disc, fossa) and the mandibular ramus. This can result in secondary vertical compensations in the maxilla and malocclusions with sagittal, transverse, and vertical implications [4]. The origin of CH may be linked to hormonal, traumatic, neoplastic, genetic, molecular, or functional factors [5].

Condylar hyperplasia (CH) presents with varying vectors of alteration, leading to distinct clinical manifestations and associated complications. Three primary forms are recognized [4,6], as detailed below.

### 1.1. Hemimandibular Elongation (HE)

This form is characterized by a horizontal vector of alteration, which shifts the mandible away from the sagittal midline. This results in consistent occlusal changes among patients. Its main etiologic factors are trauma and hormonal influences, often leading to onset during pre- and adolescence. HE also shows a higher female prevalence and is the most common form of hyperplasia.

### 1.2. Hemimandibular Hyperplasia

This type involves a vertical vector of alteration, producing three-dimensional growth on one side of the face without displacing the mandible from the mid-sagittal plane. Consequently, its occlusal characteristics differ significantly from HE. Its main etiologic factor is neoplastic growths, such as osteochondroma, and it is the least common of the hyperplasia.

### 1.3. Hybrid Form (HF)

The HF combines both horizontal and vertical vectors of alteration. This makes it a particularly severe and aggressive expression of the pathology.

Although human anatomy is not entirely symmetrical, anthropometric standards derived from population-based studies provide parametric thresholds to identify when a craniofacial asymmetry falls outside the normal range [7]. When a patient’s craniofacial anthropometric assessment (i.e., quantitative anatomical measurements) deviates from these parameters, facial asymmetry (FA) is evident, typically associated with mandibular deviation (MD) [4].

In such cases, the midpoint of the mandibular symphysis extends beyond the sagittal midplane of the cranial structures. This deviation is linked to multiple etiological factors, including genetic [8], neurological [9], neoplastic [10], molecular [11], developmental [12], environmental and traumatic [13], functional laterognathism [14], glenoid cavity asymmetry [15] and condylar resorption [16]. Such FA not only impacts bone and soft tissue structures but can also lead to alterations in extracranial structures, such as the cervical vertebrae.

The mandible and cervical spine are anatomically and functionally interconnected. This complex relationship is facilitated by shared muscle chains, including the sternocleidomastoid, suprahyoid, infrahyoid, and suboccipital muscles, as well as the hyoid bone. Furthermore, neurological connections between the trigeminal nerve and brainstem nuclei, which also regulate cervical functions, contribute to this interrelationship [17]. The C1 (Atlas) and C2 (Axis) vertebrae form the craniovertebral junction, acting as the crucial transition between the brain and the rest of the cervical spine. This area is vital as it houses neural and vascular structures while allowing for significant spinal mobility. Most vertebral rotation, flexion, and extension occur between C1 and C2, while the intervertebral discs is located from the C2-C3 level downwards [18].

Craniofacial alterations linked to FA have been thoroughly investigated, but research into extracranial alterations remains in its early stages. A crucial question in a patient’s comprehensive evaluation is how MD affects neck posture and the position of the cervical vertebrae. This is especially relevant given that some scientific literature has established an anatomical-functional and pathophysiological connection between craniomandibular and craniocervical dysfunction, integrating them into a single tonic-postural system [19].

In line with this, Guan et al. [20] and Nakashima et al. [21] have postulated an inherent correlation between MD and cervical posture, affecting it three-dimensionally. Additionally, Cardinal et al. [22] noted a positive correlation between posterior crossbite, a common feature of MD, and deviation of the first cervical vertebrae.

However, the direct correlation between specific conditions that cause FA, such as CH, and associated vertebral changes remains unexplored. Furthermore, previous studies have neither differentiated cohorts based on the severity of MD nor segmented individual vertebrae to assess their behavior comprehensively.

Given that computed tomography (CT) has proven helpful in the morphological evaluation of anatomical structures, providing detailed views of their size, shape, and position from various angles and slices [23], this study aims to evaluate the three-dimensional behavior of the upper cervical vertebrae to CH with severe MD, as observed in HE and HF.

## 2. Materials and Methods

### 2.1. Study Design and Patient Selection

We conducted a retrospective cross-sectional study, approved by the Institutional Ethics Committees of Clínica Imbanaco (CEI-856) and Universidad del Valle Faculty of Health (E 008-024). We retrieved CT datasets in DICOM format from the institutional repository, encompassing patients with a clinical diagnosis of FA who received care at Clínica Imbanaco between August 2015 and December 2023.

**Figure 1:**
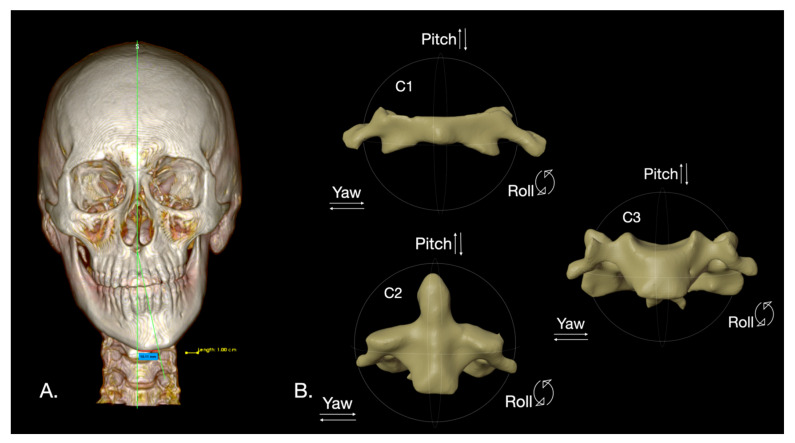
Front view. (**A**) Severe mandibular deviation (10.11 mm) to the left in a subject with condylar hyperplasia of the hybrid form. (**B**) C1, C2 and C3 vertebrae with Pitch, Roll and Yaw planes.

Our selection criteria for FA were based on the classification by López et al. [4]. We specifically chose datasets from patients with severe FA, defined as mandibular symphysis deviation of 6 mm or more from the sagittal midplane, caused by CH with a horizontal vector of alteration, such as hemimandibular elongation HE and hybrid form HF (Figure 1A).

### 2.2. CT Acquisition and Reconstruction

All CT datasets were acquired using a Biograph mCT20 PET/CT system (Siemens, Erlangen, Germany) at Clínica Imbanaco. The imaging parameters included:

- No contrast medium.
- Volume of interest: From the vertex to the sternal notch.
- Slice thickness: 0.75 mm.
- Pitch: 1.0.
- Cubic matrix: 512 × 512 with an isotropic voxel size of 0.58 mm.

CT images were reconstructed using a homogeneous low-dose B26F filter for precise anatomical localization.

### 2.3. Exclusion Criteria and Side Definition

We excluded incomplete CT datasets and those from patients with a history of:

- TMJ disease or surgery.
- Orthognathic surgery.
- Craniofacial trauma.
- Dentofacial syndrome.
- Pathologies like arthritis or other spinal diseases.

For consistency, the affected side was defined as the side with condylar overgrowth, and MD side was considered the contralateral side.

### 2.4. CT Dataset Segmentation

For each dataset, the skull, mandible, and cervical vertebrae (C1–C3) were segmented. A trained researcher manually selected hard tissue structures using the Threshold tool within the Segment Editor of the open-source software 3D Slicer (v. 5.8.1 r33241/11eaf62, USA) [24]. This selection was based on Hounsfield Units (HU) ranging from 140 HU to 5734 HU. Subsequently, a semi-automatic segmentation generated Standard Tessellation Language (.stl) files for the merged skull and mandible, as well as for each cervical vertebra (Figure 1B).

### 2.5. Cephalometric Landmark Selection

The Frankfort horizontal plane was used as the reference plane because it is defined by stable cranial landmarks that are minimally influenced by mandibular growth, asymmetry, or positional alterations. Since this study evaluates three-dimensional displacement of the upper cervical vertebrae associated with severe mandibular deviation secondary to condylar hyperplasia, a cranial-based reference system prevents mandibular displacement from being incorporated into the reference itself and allows cervical positional changes to be assessed relative to the cranial base.

**Figure 2:**
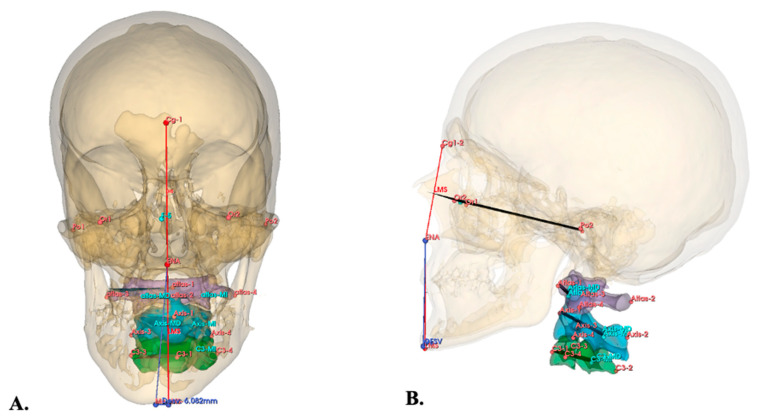
(**A**) Frontal view. Severe subject diagnosed with CH of the HF type, anatomical points located Cg1-2 (Crista Galli), ENA (Anterior Nasal Spine), LMS (Mid Sagittal Line), Me (Menton), in yellow the segmented skull is illustrated, in purple the C1 vertebra, in blue the C2 vertebra, in green the C3 vertebra, all this to evaluate the magnitude of MD and the Roll and Yaw of the cervical vertebrae. (**B**) Sagittal view. Subject with severe MD, diagnosed with CH. Anatomical points located Cg1-2 (Crista Galli), ENA (Anterior Nasal Spine), LMS (Mid Sagittal plane), Me (Menton), Or1 (Right Orbit), Or2 (Left Orbit), Po1 (Right Porion), Po2 (Left Porion), Atlas 1-2-3-4-MD-MI, Axis 1-2-3-4-MD-MI, C3 1-2-3-4-MD-MI, in yellow the segmented skull is illustrated, in purple the C1 vertebra, in blue the C2 vertebra, in green the C3 vertebra, all this to evaluate the up–down movement (pitch) of the first three cervical vertebrae.

The Frankfort plane, serving as the reference, was established by placing four primary cephalometric landmarks on the skull using the Markups module in 3D Slicer (Table 1). Following this, primary cephalometric landmarks were similarly placed on the C1 (Atlas), C2 (Axis), and C3 vertebrae to define their respective planes of interest (Table 1). To determine the three-dimensional (3D) position of each vertebra based on Pitch, Yaw, and Roll angle deviations relative to the Frankfurt plane, the Angle Planes module and the Middle Point tool in 3D Slicer were utilized. Derivative landmarks were automatically generated by the software (Table 1, Figure 2).

### 2.6. Outcomes of Interest

MD was quantified in millimeters (mm) using the method described by López et al. [4] (Figure 1). For the C1–C3 vertebrae, their 3D positions relative to the Frankfurt plane were recorded as Pitch, Yaw, and Roll angles in degrees (°) (Figure 2).

### 2.7. Statistical Analysis

A single researcher (CMR) obtained all measurements. To assess intra-examiner agreement, 10 randomly selected CT datasets were re-measured after two weeks, with agreement evaluated using the Wilcoxon signed-rank test.

Data are reported as Mean ± SD. The Shapiro–Wilk test was used to assess data normality. Samples were divided into two groups based on the side of CH (right and left). The Kruskal–Wallis test, followed by Dunn’s multiple comparisons test, was used to compare outcomes of interest between these groups. Spearman’s correlation coefficient was used to calculate the correlation between MD and the 3D position of the vertebrae.

All analyses were performed using GraphPad Prism 10 for macOS (Version 10.4.2/534). A *p*-value < 0.05 was considered statistically significant.

## 3. Results

From an initial 247 revised CT datasets, we included 50 from patients with CH. The mean age was 22 ± 7.83 years, with similar mean ages for both right-sided CH (22.5 ± 9.37 years; *n* = 28) and left-sided CH (21.6 ± 6.52 years; *n* = 22). Females predominated in the left CH group (77%), while males were more frequent in the right CH group (57%). High-frequency (HF) was more common in both groups (59% left, 61% right) compared to low-frequency (HE) (41% left, 39% right).

When comparing right versus left CH, no significant differences were found for age (*p* = 0.842), CH type (*p* > 0.999), or amount of MD (*p* = 0.729). However, there was a significant difference in patient distribution based on sex (*p* = 0.021) (Table 2).

We evaluated the lateral inclination (roll) and axial rotation (yaw) of cervical vertebrae C1, C2, and C3, classifying movements as rightward, leftward, or no movement, relative to the CH side. Although a consistent lateral Roll orientation of C2 and C3 toward the MD side was observed, it is important to distinguish between directional distribution and angular magnitude. Table 3 reports the frequency of Roll orientation toward each side and therefore reflects a significant directional pattern. In contrast, the comparison of mean Roll angles evaluates the magnitude of inclination, which did not show statistically significant differences between sides. Thus, the significant findings indicate a preferential direction of Roll rather than a greater degree of angular displacement.

For C1, the lateral roll inclination pattern showed no statistically significant difference between groups (*p* = 0.12). However, leftward inclination was more prevalent in the right CH group (54%), while rightward inclination predominated in the left CH group (45%).

At C2, we identified statistically significant differences in lateral roll inclination (*p* = 0.006). Most patients with left CH presented C2 inclination to the right (77%), aligning with the side of MD. Conversely, leftward inclination predominated in the right CH group (50%).

Significant differences were also noted for C3’s lateral roll inclination pattern (*p* = 0.045). In both the right and left CH groups, 64% of patients exhibited lateral inclination toward the side of their MD.

Regarding axial rotation (yaw) movement, no statistically significant differences were observed in any of the three evaluated vertebrae (C1: *p* = 0.7; C2: *p* = 0.4; C3: *p* = 0.2). However, there was considerable dispersion in rotation directions, with similar proportions across both groups. For example, 50% of the left CH group’s C1 vertebrae rotated to the right, compared to 39% in the right CH group.

Movement Angles (Supplementary Table S1)

Pitch (Flexion/Extension)

Mean pitch angles were similar between right and left CH groups across all three evaluated vertebrae:

- **C1**: 11.6° ± 7.26° (95% CI: 8.79–14.4°) in right CH versus 12.1° ± 5.40° (95% CI: 9.74–14.5°) in left CH.
- **C2**: Higher in left CH (18.6° ± 5.53°) than right CH (16.0° ± 6.67°), though not statistically significant (*p* > 0.05).
- **C3**: Similar between groups (5.43° in right CH vs. 4.73° in left CH; *p* > 0.05).

Yaw (Axial Rotation)

Axial rotation angles (yaw) displayed marked interindividual variability, with no significant association with the side of CH:

- **C1**: 44.0° ± 54.7° (right CH) versus 46.6° ± 57.6° (left CH).
- **C2**: 45.5° ± 54.2° (right CH) versus 59.4° ± 63.8° (left CH).
- **C3**: 45.3° ± 54.4° (right CH) versus 19.5° ± 23.3° (left CH).

Roll (Lateral Inclination)

Roll angles were low and similar between groups, with no significant differences:

- **C1**: 2.25° ± 2.49° (right CH) versus 2.41° ± 2.61° (left CH).
- **C2**: 2.18° (right CH) versus 2.32° (left CH).
- **C3**: Slightly higher in right CH (2.68° ± 1.74°) than left CH (1.91° ± 1.66°), without statistical significance.

**Figure 3:**
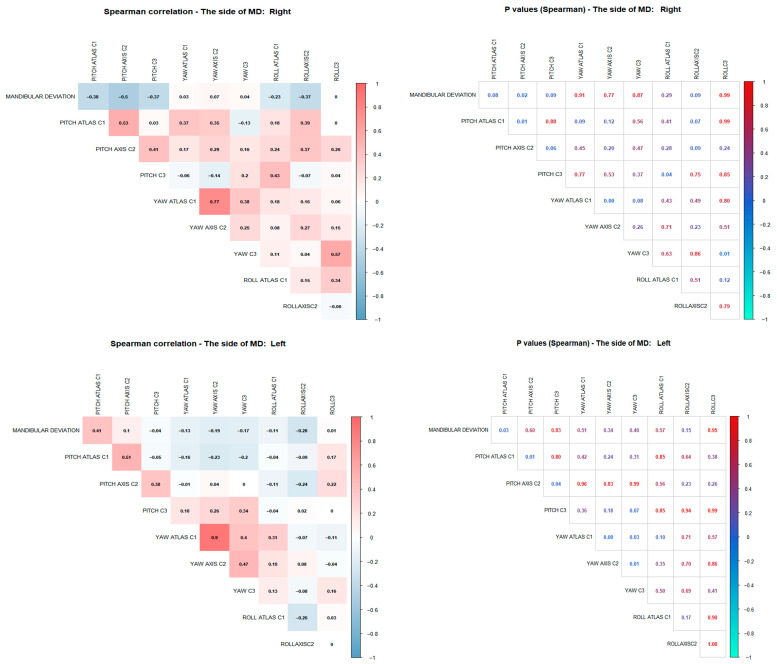
Spearman’s correlation matrix between mandibular deviation and the three-dimensional movements of the cervical vertebrae (Pitch, Yaw, and Roll), differentiated by side of the deviation. statistically significant (Spearman’s correlation coefficient). Abbreviations: MD, mandibular deviation; r, correlation coefficient.

A Spearman’s correlation analysis investigated the relationship between MD magnitude (mm) and the 3D motion angles (Pitch, Yaw, and Roll) of cervical vertebrae C1, C2, and C3. The results were analyzed independently for each side of MD (Figure 3). Because several correlations were explored, the statistical analysis was designed as exploratory and hypothesis-generating. Accordingly, no adjustment for multiple comparisons was applied, and findings are intended to inform future confirmatory studies.

For patients with rightward MD, there was a significant negative correlation between MD and C2 Pitch (r = −0.50; *p* = 0.02). A non-significant negative correlation was also noted with C1 Pitch (r = −0.38; *p* = 0.08). These findings suggest that greater leftward MD correlates with lower pitch angles in both C1 and C2.

In patients with leftward MD, a significant positive correlation was observed between MD magnitude and C1 Pitch angle (r = 0.41; *p* = 0.03). Conversely, a significant negative correlation was found with C2 Pitch (r = −0.51; *p* = 0.01). This indicates that greater rightward MD is associated with increased C1 pitch and decreased C2 pitch.

Relevant Intravertebral Correlations (Figure 3)

Analysis of correlations between movement planes within each vertebra revealed strong and statistically significant associations, especially in the Atlas (C1).

In both right and left MD patient groups, we observed a strong positive correlation between the Pitch and Yaw angles of C1 (r = 0.77, *p* < 0.001 in the right group; r = 0.90, *p* < 0.001 in the left group). This indicates a high degree of synchrony between vertical and rotational movements at this level.

Similarly, in patients with left MD, a significant positive correlation was found between the Pitch and Yaw angles of the Axis (C2) (r = 0.47, *p* = 0.01), suggesting that, as in C1, the Axis may also exhibit coordinated movement as part of a superior cervical adaptive pattern in response to MD (Figure 3). Additionally, in the group with right deviation, a significant correlation was identified between Yaw and Roll in the C3 vertebra (r = 0.57, *p* = 0.01). (Figure 3).

## 4. Discussion

This study utilized computed tomography to analyze craniofacial structure and MD in patients with CH. Through tomographic segmentation, we individualized the cervical vertebrae to assess their three-dimensional behavior relative to the hyperplasia. Our results indicate changes in vertebral inclinations, predominantly towards the contralateral side of the hyperplasia—the side of mandibular deviation. These findings provide a comprehensive understanding of how craniofacial alterations can manifest in extracranial structures, such as the cervical vertebrae, potentially as adaptive responses or due to altered mechanical loads.

Our research sample consisted of patients diagnosed with unilateral CH. This condition involves disproportionate growth of one condyle compared to the other, leading to not only FA but also significant articular and occlusal changes that affect the entire maxillomandibular system [6]. While unilateral CH presents in three forms, this study focused exclusively on HE and HF. These are the most common CH entities and share a horizontal component in their alteration, effectively displacing the mandible away from the sagittal midplane. We included only severe cases, defined as those with MD of 6 mm or more. This threshold signifies deviations that typically exceed personal acceptance and social perception, as reported by Wang et al. [7], and commonly necessitate orthodontic or surgical correction.

In our sample, 58% of severe unilateral CH cases occurred in women. This aligns with numerous studies and systematic reviews, including that of Raijmakers et al. [25], which suggest a higher female predisposition, potentially considering female gender a risk factor for unilateral CH. This predisposition, along with other TMJ disorders, may be linked to hormonal differences, particularly estrogen, which regulates bone growth and is evidenced in the TMJ [26]. This study also revealed a predominance of right condyle alteration (56%), leading to left-sided MD (mandibular levognathism), an incidence also reported by Lopez et al. [27].

Regarding the relationship between CH, MD, and vertebral inclinations, our study demonstrated that the lateral roll inclination for both C2 and C3 vertebrae was significantly (*p* < 0.05) more pronounced towards the side contralateral to the CH, which corresponds to the side of MD.

Similar findings were reported by Cardinal et al. [22], who observed lateral roll inclination of C2 and C3 on the side of MD and unilateral posterior crossbite in a 26-patient sample analyzed via CBCT. Expanding on these observations, Guan et al. [20] conducted a CBCT study on 30 subjects with MD, comparing them to individuals without FA to analyze 3D cervical vertebral changes. They concluded that a correlation exists between MD and vertebral deflection, which influences the 3D position of the cervical vertebrae. Despite methodological differences—Guan et al. [20] included asymmetric cases from 2 mm of MD, while our study focused solely on severe cases from 6 mm—the results align. Therefore, as stated by Lippold et al. [28], these studies collectively provide evidence of a relationship between jaw position and upper spinal body posture.

It is important to note that MD not only affects facial aesthetics and produces differential joint loading but also leads to asymmetrical occlusal changes, often resulting in crossbite on the deviating side [3,5]. In this regard, Matsaberidze et al. [19] theorized that inadequate occlusion can cause misalignment of the upper cervical vertebrae, leading to skull tilting and deviation from a symmetrical and balanced position above the shoulders. This, in turn, can sometimes result in postural collapse that affects, strains, and compresses the brainstem, thereby suggesting a relationship between occlusion and posture.

This theory aligns with various research findings. For instance, Meibodi et al. [29], Sonnesen et al. [30], and Aranitasi et al. [31] found a significant correlation between cervical vertebrae anomalies and deviations with skeletal malocclusions, such as Class III. Furthermore, studies like Korbmacher et al. [32] have demonstrated an association between unilateral crossbite and cervical orthopedic disorders.

The above findings suggest a possible association between MD and 3D characteristics of the cervical vertebrae; however, the clinical relevance of these changes for craniocervical anatomy remains uncertain. Previous studies have explored potential links between mandibular position and postural behavior. For example, Wakano et al. [33] reported that horizontal deviations in jaw position may be associated with alterations in postural stability, while Ben-Bassat et al. [34] described a higher prevalence of asymmetric malocclusion features in patients with idiopathic scoliosis. Likewise, Ozturk et al. [35] reported relationships between the cranio-cervico-mandibular system and temporomandibular disorders. These reports provide contextual background but do not establish direct causal pathways between malocclusion and global postural alterations.

In the present study, no statistically significant differences were observed in vertebral anthropometric measurements between groups based on the CH side or MD side across any of the evaluated planes (Pitch, Yaw, or Roll). However, the high dispersion of Yaw values, particularly in C1 and C2, is notable. This might reflect greater individual variability in the axial rotation of these vertebrae in patients with severe MD, possibly due to the wide range of flexion, extension, and rotational movements inherent to these cervical segments [36].

Regarding Pitch (up–down) motion, we observed a significant decrease in the C2 angle for both sides of MD (*p* < 0.05). This means greater MD correlates with a more pronounced downward motion at C2.

Our correlation analysis of motion planes within each vertebra revealed strong, statistically significant associations, especially in the Atlas (C1). This indicates high synchrony between C1’s vertical (Pitch) and rotational (Yaw) movements, suggesting an integrated functional response of C1 to postural changes linked to MD.

Similarly, in the left CH and right MD group, a significant positive correlation between Pitch and Yaw was found in the Axis (C2) vertebra (r = 0.47, *p* = 0.01). Like C1, C2 appears to participate coordinately in cervical adaptation to MD. This finding may reflect a functional coupling in the upper cervical spine, possibly associated with compensatory mechanisms or stabilization of the postural axis in relation to MD. This is plausible given that the craniovertebral junction—the complex transition between the brain and cervical spine involving the atlas and axis joined by the odontoid apophysis—houses vital neural and vascular structures while allowing for the greatest range of spinal movements [17,18].

The 3D positional deviations observed at C1, C2, and C3 in the present study may reflect a neuromuscular compensatory response to MD associated with CH. However, due to the cross-sectional design of this investigation, the absence of a non-asymmetrical control group, and the limited body of literature addressing this specific interaction, a definitive cause-and-effect relationship between MD and cervical spine positioning cannot be established.

Although existing theoretical frameworks suggest that the stomatognathic system may influence cervical alignment, its overall contribution to global postural control remains insufficiently understood. Moreover, without comparison to individuals presenting mandibular asymmetry of non-pathological origin, it remains uncertain whether the observed alterations in cervical vertebral angulation exceed normal anatomical variability or represent adaptive postural responses.

Accordingly, these findings should be interpreted as exploratory and hypothesis-generating, as it remains uncertain whether the observed changes in vertebral angulation fall within normal anatomical variability or represent postural alterations with potential clinical relevance. In addition, the synchronized Pitch–Yaw behavior observed at C1 and C2 may reflect different, non-exclusive mechanisms, including biomechanical adaptation to maintain cranio-cervical balance, neuromuscular compensation aimed at preserving head orientation, or simply a coincidental anatomical association in the absence of a control group. Future prospective and longitudinal studies, incorporating both symmetrical healthy controls and appropriately matched asymmetrical groups, are required to elucidate the temporal and causal relationships between mandibular deviation, occlusal characteristics, cervical spine alignment, and overall body posture, as well as their potential implications for orthodontic diagnosis, orthognathic surgical planning, and postural assessment.

Study Limitations: This study has several limitations. First, its retrospective design involves an inherent risk of selection bias, as cases were included based on the availability of imaging records rather than predefined sampling criteria. Second, the absence of a control group of subjects with facial symmetry limits the ability to establish causal relationships between condylar hyperplasia, mandibular deviation, and upper cervical vertebral displacement. Therefore, the present results should be interpreted as descriptive and associative rather than causal.

In addition, although three-dimensional spatial changes were quantified, potential confounding variables were not controlled. Future investigations should adopt prospective designs including age- and sex-matched control groups to better isolate the effect of condylar hyperplasia on upper cervical morphology and orientation and to improve the generalizability of the findings.

## 5. Conclusions

The key findings from this study are:

- Vertebral Lateral Inclination: Patients with CH exhibiting a horizontal alteration vector (HE and HF) show a greater proportion of lateral Roll inclination in their C1, C2, and C3 vertebrae. This inclination is consistently directed towards the contralateral side of the hyperplasia, which is also the side of MD.
- Mandibular Deviation and C2 Pitch: As the magnitude of MD increases (for both right and left deviation), the Pitch of the C2 vertebra becomes more negative. This indicates a downward, or inferior, movement of the C2 vertebra.
- Synchronized Upper Cervical Movement: There is a high degree of synchrony between Pitch and Yaw movements in both the C1 and C2 vertebrae. This suggests that these vertebrae exhibit coordinated motion, likely as part of an adaptive pattern in response to MD.

## Figures and Tables

**Table 1:** Description of the cephalometric landmarks used to determine the reference plane on the skull and the planes of interest in the cervical vertebrae.

STRUCTURE	PRIMARY	DESCRIPTION	ABBREVIATION
**SKULL**	Porion (Po)	Most superior point of the margin of the external acoustic meatus.	Po1: Right Porion Po2: Left Porion
	Orbitale (Or)	Most inferior point on the margin of the orbit.	Or1: Right Orbitale Or2: Left Orbitale
	**DERIVATE**	**DESCRIPTION**	**ABBREVIATION**
	F5	Middle Point between Or1 and Or2.	F5
**CERVICAL VERTEBRAE**	**PRIMARY**	**DESCRIPTION**	**ABBREVIATION**
	Point 1	The most prominent part of the anterior tubercle.	C1-1; C2-1; C3-1
	Point 2	The most prominent part of the posterior tubercle.	C1-2; C2-2; C3-2
	Point 3	The most lateral edge of the right transverse process.	C1-3; C2-3; C3-3
	Point 4	The most lateral edge of the left transverse process.	C1-4; C2-4; C3-4
	**DERIVATE**	**DESCRIPTION**	**ABBREVIATION**
	Medium Right	The midpoint between Point 2 (posterior tubercle) and Point 3 (right transverse process)	MR
	Medium Left	The midpoint between Point 2 (posterior tubercle) and Point 4 (left transverse process).	ML

**Table 2:** Sample distribution according to age, sex, condylar hyperplasia type, and affected side.

	Affected Side	
	Right (28)	Left (22)
**Age** Median ± SD (95% CI)	21.6 ± 6.52 (19.1–24.1)	22.5 ± 9.37 (18.3–26.6)
**Sex**		
Female	12 (43%)	17 (77%)
Male	16 (57%)	5 (23%)
**Diagnosis**		
HE	11 (39%)	9 (41%)
HF	17 (61%)	13 (59%)
**MD in mm** Median ± SD (95% CI)	7.43 ± 1.37 (6.90–7.96)	7.39 ± 1.75 (6.60–8.15)

To compare the groups based on the age and the amount of MD, the Mann–Whitney test was used, whereas, to compare the groups based on the distribution of the sex and the CH types, the Chi-square test (Fisher’s exact test) was used. Abbreviations: MD, mandibular deviation; HF, hybrid form; HE, hemimandibular elongation.

**Table 3:** Distribution of the direction of the Roll and Yaw movements of the cervical vertebrae (C1–C3) according to the side of the CH.

	Affected Side		
Variable	Right (28)	Left (22)	*p*-Value
**Inclination Roll C1 Atlas (right/left)**			0.12
Right	5 (18%)	10 (45%)	
Left	15 (54%)	8 (36%)	
No inclination	8 (29%)	4 (18%)	
**Inclination Roll C2 Axis (right/left)**			**0.006**
Right	10 (36%)	17 (77%)	
Left	14 (50%)	5 (23%)	
No inclination	4 (14%)	0 (0%)	
**Inclination Roll C3 (right/left)**			**0.045**
Right	9 (32%)	14 (64%)	
Left	18 (64%)	8 (36%)	
No inclination	1 (4%)	0 (0%)	
**Rotation Yaw C1 Atlas (right/left)**			0.7
Right	11 (39%)	11 (50%)	
Left	8 (29%)	6 (27%)	
No rotation	9 (32%)	5 (23%)	
**Rotation Yaw C2 Axis (right/left)**			0.4
Right	17 (61%)	9 (41%)	
Left	9 (32%)	10 (45%)	
No rotation	2 (7%)	3 (14%)	
**Rotation Yaw C3 (right/left)**			0.2
Right	17 (61%)	10 (45%)	
Left	9 (32%)	12 (55%)	
No rotation	2 (7%)	0 (0%)	

The bold format of *p*-value indicates that *p* < 0.05.

## Data Availability

Data are available upon request due to restrictions, such as privacy or ethics.

## Supplementary Materials

The following supporting information can be downloaded at: https://www.mdpi.com/article/10.3390/diagnostics16040579/s1, Table S1: Mean values of the three-dimensional angles (Pitch, Yaw and Roll) of the cervical vertebrae according to the CH side.

## Abbreviations

The following abbreviations are used in this manuscript:

MD	Mandibular deviation
CH	Condylar hyperplasia
CT	Computed Tomography
HE	Hemimandibular elongation
HF	Hybrid form
TMJ	Temporomandibular joint
FA	Facial asymmetry
C1	Atlas
C2	Axis
