# Supplementary material for: Three-Dimensional Displacement of Upper Cervical Vertebrae in Severe Mandibular Deviation Caused by Condylar Hyperplasia: A Tomographic Segmentation Study
Source: Diagnostics (Basel). 2026 Feb 14;16(4):579. doi: 10.3390/diagnostics16040579 (PMC12939729; doi:10.3390/diagnostics16040579)
Supplement: Supplementary file 1 [file diagnostics-16-00579-s001.zip › diagnostics-4127940-supplementary.pdf]

Supplementary Table 1. Movement Angles.

General Data (50)					Affected side					
					Right (28)			Left (22)		
**Variable**	**Median ± DS**	**CI (95%)**	**Min**	**Max**	**Median ± DS**	**95% CI**	**Min-Max**	**Median ± DS**	**95% CI**	**Min-Max**
**Pitch Atlas C1**	11.8 ± 6.45	(10.0 – 13.7)	1	27	11.6 ± 7.26	(8.79-14.4)	1-27	12.1 ± 5.40	(9.74-14.5)	2-21
**Pitch Atlas C2**	17.2 ± 6.27	(15.4 – 18.9)	2	33	16.0 ± 6.67	(13.3-18.6)	2-32	18.6 ± 5.53	(16.1-21.0)	8-33
**Pitch C3**	5.12 ± 4.42	(3.86 - 6.38)	0	17	5.43 ± 4.38	(3.73-7.13)	0-15	4.73 ± 4.55	(2.71-6.74)	1-17
**Roll Atlas C1**	2.32 ± 2.52	(1.60 – 3.04)	0	9	2.25 ± 2.49	(1.28-3.22)	0-9	2.41 ± 2.61	(1.25-3.57)	0-9
**Roll Axis C2**	2.24 ± 1.86	(1.71 – 2.77)	0	8	2.18 ± 1.93	(1.43-2.93)	0-8	2.32 ± 1.81	(1.52-3.12)	0-6
**Roll C3**	2.34 ± 1.73	(1.85 – 2.83)	0	6	2.68 ± 1.74	(2.00-3.35)	0-6	1.91 ± 1.66	(1.17-2.64)	0-5
**Yaw Atlas C1**	45.2 ± 55.4	(29.4 – 60.9)	0	179	44.0 ± 54.2	(22.8-65.2)	0-172	46.6 ± 57.6	(21.1-72.2)	0-179
**Yaw Axis C2**	51.6 ± 58.4	(35.0 – 68.2)	1	172	45.5 ± 54.2	(24.5-66.6)	2-169	59.4 ± 63.8	(31.1-87.7)	1-172
**Yaw C3**	34.0 ± 45.1	(21.2 – 46.8)	0	170	45.3 ± 54.4	(24.2-66.4)	0-170	19.5 ± 23.3	(9.20-29.9)	0-99
